# Computational Modelling Enabling In Silico Trials for Cardiac Physiologic Pacing

**DOI:** 10.1007/s12265-023-10453-y

**Published:** 2023-10-23

**Authors:** Marina Strocchi, Nadeev Wijesuriya, Vishal Mehta, Felicity de Vere, Christopher A. Rinaldi, Steven A. Niederer

**Affiliations:** 1 National Heart and Lung Institute, Imperial College London, 72 Du Cane Road, W12 0HS, London, UK; 2 School of Biomedical Engineering and Imaging Sciences, King’s College London, London, UK; 3 Guy’s and St Thomas’ NHS Foundation Trust, London, UK; 4 The Alan Turing Institute, London, UK

**Keywords:** Cardiac resynchronization therapy, His bundle pacing, Left bundle pacing, Conduction system pacing, In silico, In silico trials, Modelling

## Abstract

Conduction system pacing (CSP) has the potential to achieve physiological-paced activation by pacing the ventricular conduction system. Before CSP is adopted in standard clinical practice, large, randomised, and multi-centre trials are required to investigate CSP safety and efficacy compared to standard biventricular pacing (BVP). Furthermore, there are unanswered questions about pacing thresholds required to achieve optimal pacing delivery while preventing device battery draining, and about which patient groups are more likely to benefit from CSP rather than BVP. In silico studies have been increasingly used to investigate mechanisms underlying changes in cardiac function in response to pathologies and treatment. In the context of CSP, they have been used to improve our understanding of conduction system capture to optimise CSP delivery and battery life, and noninvasively compare different pacing methods on different patient groups. In this review, we discuss the in silico studies published to date investigating different aspects of CSP delivery.

**Graphical Abstract:**

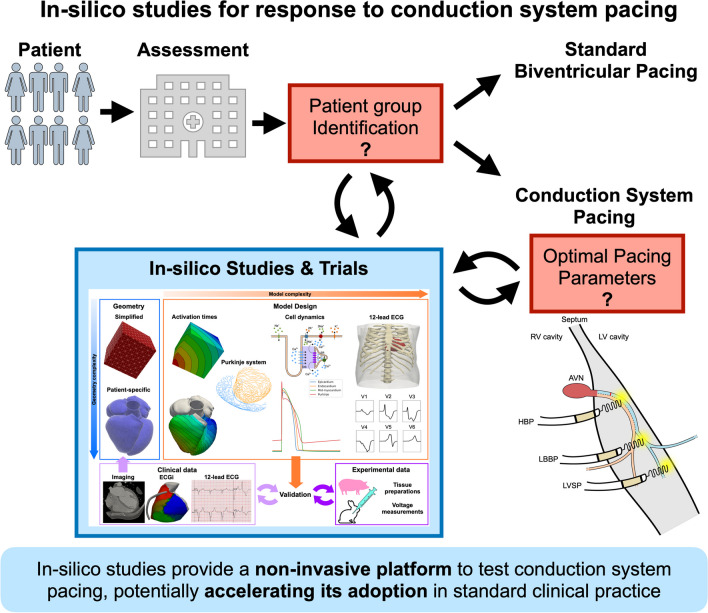

**Supplementary Information:**

The online version contains supplementary material available at 10.1007/s12265-023-10453-y.

## Cardiac Resynchronisation Therapy and Conduction System Pacing

**Fig. 1:**
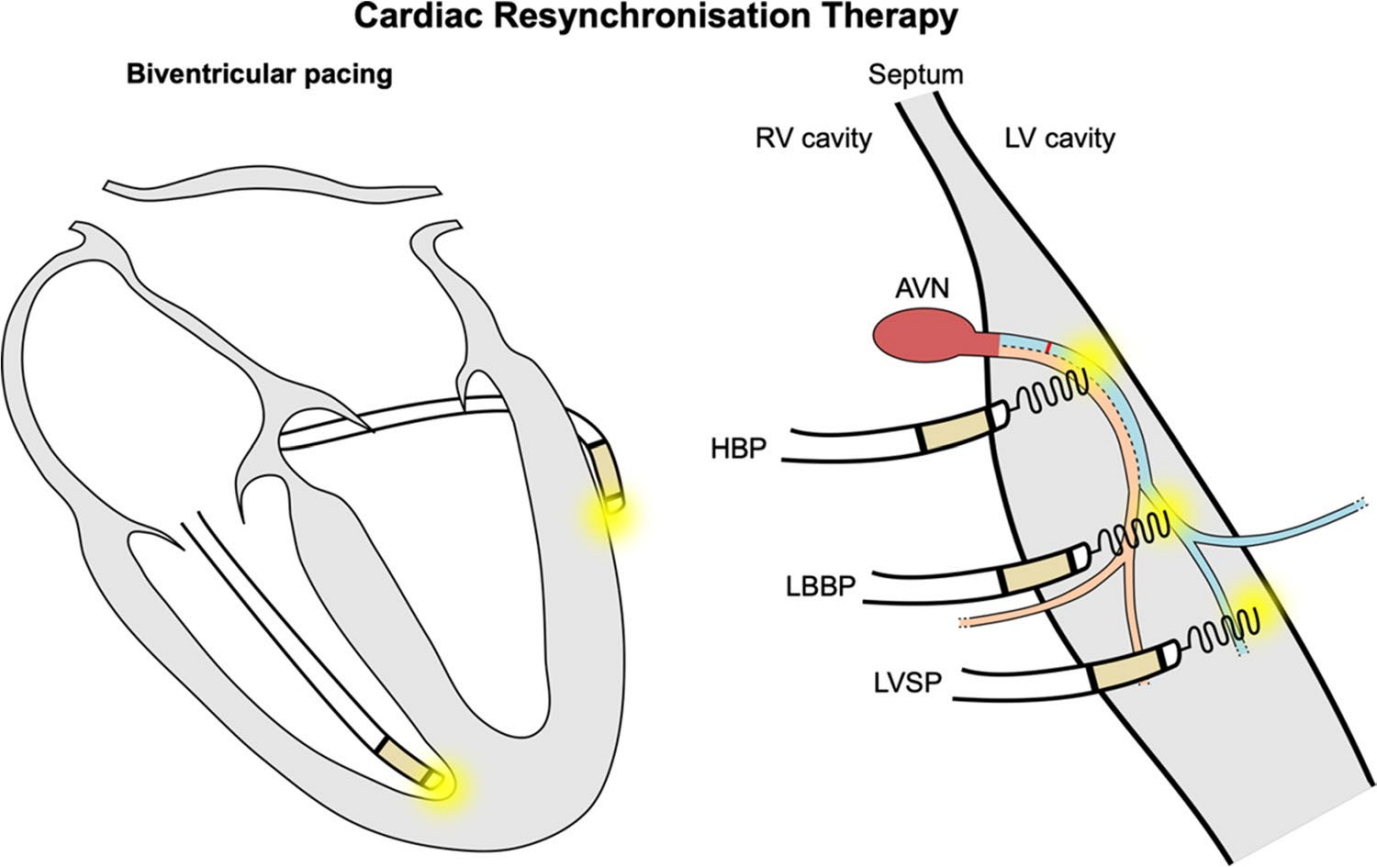
Cardiac resynchronisation therapy delivery methods. Biventricular pacing (left) is delivered by pacing the RV apex and the LV epicardium from a tributary of the coronary sinus. Conduction system pacing can be delivered by implanting a lead in the His (HBP) or in the left bundle branch area (LBBAP) from the RV side. When the left bundle is directly targeted, LBBP is achieved. On the other hand, LV septal pacing (LVSP) is delivered by pacing the myocardium of the deep intraventricular septum without directly targeting the left bundle

Cardiac resynchronisation therapy (CRT) is an effective treatment for dyssynchronous heart failure patients, aiming to restore synchrony and to ultimately improve cardiac function [1]. CRT is delivered through biventricular pacing (BVP), consisting of a right ventricular (RV) lead (normally located at the RV apex) and a left ventricular (LV) lead implanted in one of the coronary sinus tributaries (Fig. 1, left). CRT is indicated in patients with left bundle branch block (LBBB) with a QRS duration (QRSd) ≥ 130 ms and reduced LV ejection fraction (LVEF ≤ 35%). Patients with non-LBBB morphology are indicated for implant if they present a broad QRS > 150 ms [2].

BVP has been shown to reduce heart failure hospitalisation, symptoms, mortality and to induce reverse remodelling, especially in patients with LBBB [3, 4]. On the other hand, evidence of BVP benefits for non-LBBB patients is limited [4, 5]. Large multi-centre clinical trials still report between 30 and 50% of non-responders [6], defined as those patients who, despite optimal drug therapy and CRT implantation, do not experience clinical improvement. This has been attributed to different factors, including sub-optimal LV lead placement, intricated coronary sinus anatomy and presence of scar [6]. Due to the heterogenous population, the cause of lack of response is likely different in each individual patient, demanding both a personalised approach to CRT and new methods to deliver cardiac physiologic pacing beyond BVP.

## Conduction System Pacing

Conduction system pacing (CSP) is emerging as a novel method to deliver pacing [7]. CSP is delivered either through His bundle pacing (HBP) or left bundle branch area pacing (LBBAP) by pacing the His or by targeting the proximal left bundle and/or the surrounding myocardium, respectively (Fig. 1, right). During BVP, the LV is often activated from the LV free wall to the septum, leading to an unphysiological activation in the opposite direction to normal activation during sinus rhythm. In contrast, CSP has the potential to restore LV physiological activation by pacing the native conduction system [7]. HBP can be delivered either selectively or non-selectively, where the His is captured alone or together with the surrounding myocardium, respectively. During LBBAP, the depth of the lead within the intra-ventricular septum determines selective capture, non-selective capture or LV septal pacing, where only the septal myocardium is paced without left bundle capture [7]. Although selective pacing is preferable, most CSP implants result in non-selective pacing [8]. It is, however, unknown if selective vs non-selective CSP significantly affects response [7, 9–11]. Compared to LBBAP, HBP is difficult to perform as the His bundle is a smaller target area than the left bundle and often requires high pacing thresholds to achieve selective pacing, leading to concerns about pacemaker battery longevity and success rates in non-specialised centres [12].

In patients where CSP alone does not restore LV synchrony, CSP can be delivered in conjunction with a LV epicardial lead to deliver His-optimised CRT (HOT-CRT) or LBBP-optimised CRT (LOT-CRT) [13, 14]. While the feasibility and safety of HOT-CRT and LOT-CRT has been proven in a clinical setting, there are still questions about which patient groups are likely to benefit from these rather than CSP or CRT alone.

As mentioned above, LBBAP is an attractive alternative to HBP because it is easier to perform and may achieve lower and more stable pacing thresholds and better sensing [15]. However, LBBAP is associated with non-negligible risks, including septal perforation, thrombus formation and coronary artery injury [16]. Importantly, the long-term outcomes of LBBAP are yet to be determined, particularly in relation to revisions or extractions of such a deep-seated lead. Possible lead-related issues may be subverted by delivering LBBAP with a leadless pacing system [17]. The WiSE-CRT system (EBR Systems, Sunnyvale, CA) is the only commercially available system to deliver leadless pacing in the LV [18]. It requires a pre-existing RV pacing co-implant and is constituted by a leadless electrode and an ultrasound transmitter implanted between the patient’s ribs. The transmitter senses the RV pacing spike and communicates with the leadless electrode, which is able to convert ultrasound waves to electrical energy to pace the LV. The receiving electrode is completely endothelialised within 4 weeks, significantly reducing the risk of stroke [19]. While the leadless electrode is traditionally implanted in the LV free wall, the WiSE-CRT system has been proven safe and effective to deliver leadless LBBAP as well [20, 21]. Although more studies are required to investigate long-term complications, leadless LBBAP represents an inviting alternative to reduce the risks associated with lead-based systems, especially if combined with a leadless RV pacing device to deliver completely leadless pacing [22].

Both HBP and LBBAP have the potential to restore electrical synchrony. A growing catalogue of CSP delivery options is available. However, randomised studies with large patient numbers are required to investigate their safety and efficacy and to identify which patients are most likely to benefit from conventional BVP and novel CSP delivery methods.

## In Silico Trials for Conduction System Pacing

In silico studies are increasingly employed to improve our understanding of cardiac physiology, pathophysiology and patient’s response to treatment [23]. Once validated against clinical and/or experimental human or animal data, computational models can be used to simulate different pathologies and treatments on a single patient (in silico studies) or a group of patients (in silico trials). In silico studies and trials provide a rapid and low-cost option to explore new therapies before trials have been completed and can be used to tailor trial design and patient selection.

**Fig. 2:**
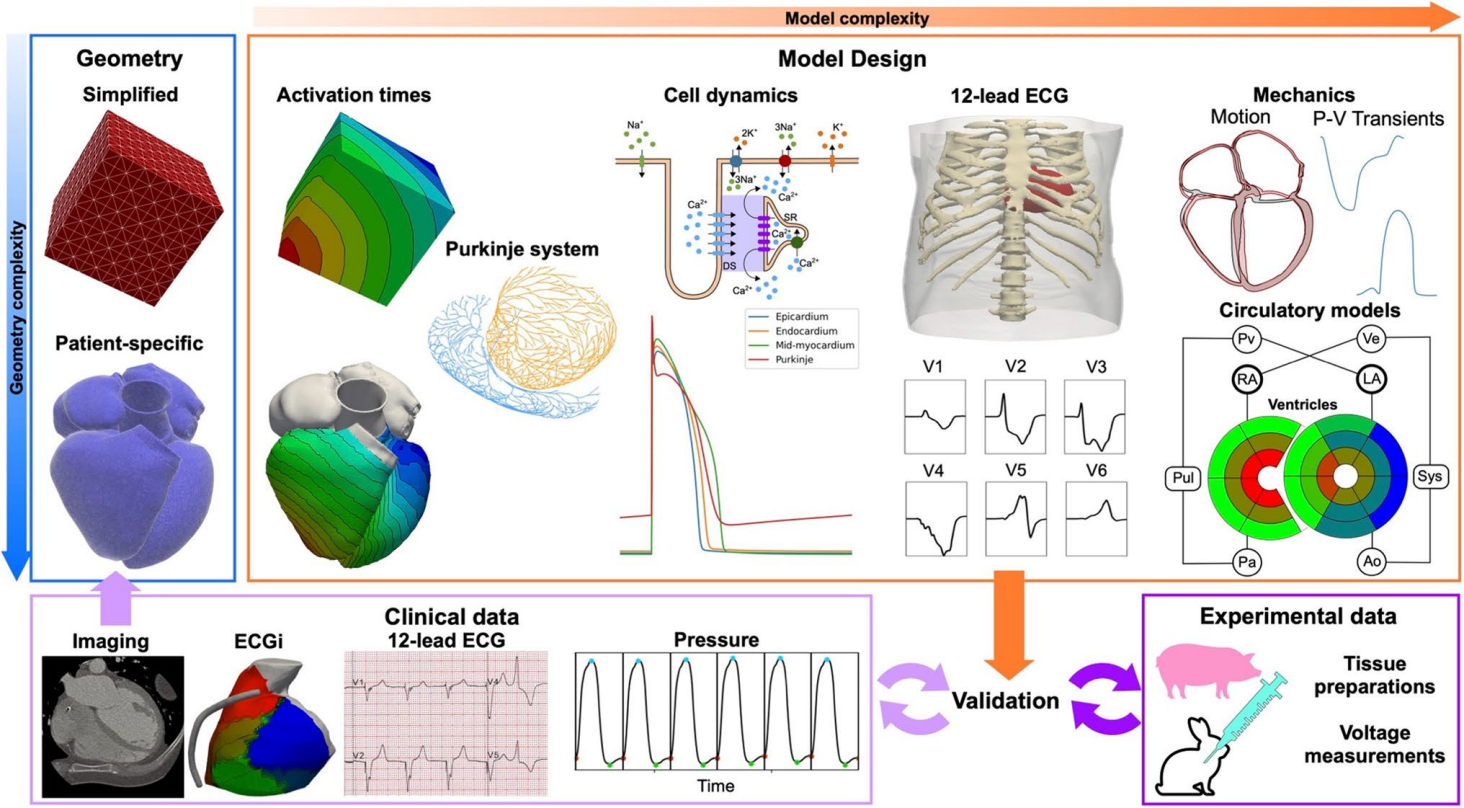
In-silico studies pipeline. The geometry can be simplified or patient-specific, normally generated by imaging data. Activation times can be simulated using an Eikonal model. Then, depending on the required complexity, a His-Purkinje system can be added. The action potential can be coupled with a monodomain or a bidomain model to simulate propagation within cardiac tissue. Finally, a torso geometry can be used to simulate a 12-lead ECG. Pressure-volume relationships can be obtained by coupling the electrophysiology model with a circulatory system or a three-dimensional mechanics model, which provides cardiac motion as well. Using clinical or experimental data, the model can then be validated to ensure model predictions are accurate for the application of interest

CRT has been studied extensively using electrical and mechanical models [24–27]. Cardiac computational models for CRT and/or CSP are built using a pipeline of image processing, meshing and calibration tools (Fig. 2). Depending on the application, the geometry can be simplified, for instance, a cube representing a tissue sample or patient-specific, segmented from computed tomography, magnetic resonance, or ultrasound imaging data. The resulting geometry is then used to run simulations to compute activation times, action potentials, propagation of the electrical signals in cardiac tissue and the torso to derive 12-lead ECGs. The electrophysiology model can also be coupled to a three-dimensional mechanics model to simulate cardiac motion or to a lumped parameter model to simulate the circulatory system. The electrophysiology and the mechanics models are finally validated against available clinical or experimental data to ensure the validity of model predictions. These modelling pipelines are now being applied to perform in silico trials for CSP to investigate optimal lead position for CSP, compare different CSP modalities, and to identify which patients are likely to derive most benefits from CSP. Most computational model employed to investigate CSP focus on electrophysiology, with only two in silico studies accounting for mechanical synchrony as well. Despite that favourable acute electrical response has been correlated to long-term patient benefits [28], improved ventricular electrical synchrony is not the sole determinant of long-term response to pacing [29–31]. In some cases, it might therefore be important for in-silico models to simulate mechanics and/or haemodynamics as well. Below, we provide a summary of the computational studies investigating different aspects of CSP delivery, highlighting the strength and weaknesses of each modelling approach. A table summarising the computational modelling studies included in this review is also provided in the Supplement Table 1.

## Lead Positioning for Conduction System Pacing Delivery

Testing the optimal position of the pacing lead for CRT delivery in vivo is challenging, as it requires increased fluoroscopy and procedure times, leading to increased patient risk [32, 33]. Furthermore, optimal lead placement might be hindered by the patient anatomy or co-existing pathologies such as the presence of scar. In the context of lead placement for CSP, the correct positioning for the pacing lead for HBP delivery is fundamental to ensure His bundle capture at low and stable pacing thresholds to optimise device battery longevity and pacing-induced synchrony. However, in clinical practice, His bundle capture often comes at the cost of high pacing thresholds and/or non-selective pacing [34]. The mechanisms underlying these issues, how these issues can be overcome or how they affect patient outcome are not completely understood. In this context, computational modelling can help understand the factors affecting the type of capture, the interaction of the lead with cardiac tissue, and to investigate how lead technology can be adapted to improve HBP delivery while maintaining low pacing thresholds.

Vigmond et al. [35] used a cardiac electrophysiology model to investigate how different factors affect His bundle capture during HBP. In this study, a simplified geometry was used to simulate a His fibre embedded in ventricular myocardium, in contact with the RV blood pool. The bidomain equations [36], the most accurate model to represent electrical propagation in the cardiac tissue, were used in conjunction with detailed ionic models for ventricular and Purkinje action potentials. This model was used to compute myocardial and His capture thresholds for different combinations of lead depth, angle w.r.t. the His fibre, polarity (anodal vs cathodal) and pulse duration. The authors observed that the myocardium was easier to capture compared to the His fibre, with even small distances between the tip of the electrode and the His leading to significantly increased His capture thresholds. Simulation results also showed that anodal capture lowered His capture thresholds, more likely leading to selective rather than non-selective pacing. As expected, increased pulse duration led to smaller capture thresholds, but, as pointed out by the authors, this would not be desirable in a clinical setting due to significantly increased energy consumption and therefore issues related to the battery life of the device. This study used a simplified geometry, and the simulation results were not validated against experimental or clinical data. Furthermore, the authors did not consider reduced His conductivities, which might occur in some pathological hearts. Nevertheless, the results helped understanding the mechanisms through which different delivery factors affect the type of capture during HBP. Furthermore, the authors highlight how a small distance of the lead tip from the His causes increased pacing thresholds. This is consistent with reported clinical experience of HBP, where difficulties to achieve selective capture have been related, among other factors, to the challenge of targeting a small area around the His [37].

The effect of HBP lead placement on conduction system capture was also investigated by Barone et al. [38] in a human biventricular model embedded in a torso geometry. The authors simulated electrical activation with a monodomain model and used the resulting activation times to compute 12-lead ECGs. The His-Purkinje system was constructed using an open-source fractal tree code [39], and action potential dynamics and heterogeneities in action potential duration from endocardium to epicardium were accounted for in the model. Healthy activation, complete AV block, complete and incomplete LBBB were simulated. The lead angle w.r.t to the His and the length of its helix were changed to investigate the effect of pacing on activation for different AV delays, pulse amplitudes and pulse durations. The model made use of ex vivo experimental data of voltage waveforms measured in four swine hearts to simulate CSP. First, the pacing protocols were simulated in a simplified geometry of the His, left and right bundles embedded in a slab of ventricular myocardium, and the delay between the terminal points of the bundles was computed for each pathology and pacing protocol. Then, the left and right bundle delays were used to simulate ventricular activation in a biventricular model, and in silico 12-lead ECGs were used to make considerations about the effect of pacing on ventricular synchrony. The ECGs simulated during LBBB were qualitatively validated against known ECG features observed in LBBB patients, but a quantitative model validation for activation times and response to pacing was lacking. The authors demonstrated that by minimising the delay between the bundles, the simulated ECGs get close to the healthy case, meaning that ventricular synchrony is restored. They considered LBBB and complete AV block only, assuming that correcting these conduction disturbances would lead to a healthy ECG. However, this might not be the case in patients where additional conduction disturbances are present and masked by proximal disease. The results presented in this in silico study also showed that, ideally, the HBP electrode should be implanted perpendicularly to the His. In order to achieve similar synchrony with an angled position, AV delay optimisation or increased pacing thresholds might be required. The authors also speculate that a longer helix lead design might help in this scenario, making selective capture of the His easier. Despite the lack of quantitative model validation, this in silico study further elucidates mechanisms through which different pacing factors affect response to HBP. Furthermore, the use of 12-lead ECG and a human model make these findings more directly applicable to a clinical context.

## Comparison Between Different Pacing Modalities

Randomised clinical trials provide a reliable source of information of how patients respond to different therapies. However, clinical trials for CRT delivery often include very heterogeneous patient populations, potentially leading to inconsistent results between studies. Furthermore, in vivo data collection can require invasive and lengthy procedures that can increase risks to patients. In this context, in silico studies can be used to test different pacing modalities under well-defined pathological conditions and explicitly stated assumptions and can therefore help us understand the mechanisms underlying response, consequently improving heart failure patient stratification and potentially informing clinical trial design.

Computational electrophysiology has been used to help understand the mechanisms behind pacing-induced ventricular synchrony during LBBP with and without AV delay optimisation [40, 41]. In 2020, in an in silico trial by Strocchi et al. [41], twenty-four patient-specific anatomical models [42] were used to simulate ventricular activation during proximal LBBB and different pacing modalities. Ventricular activation times were computed using a reaction-Eikonal model [43], where the activation times provided by the Eikonal models are used to locally trigger activation of the cell, with ventricular action potential dynamics represented with a detailed ventricular ionic model. Tissue propagation across the ventricles was then used to compute 12-lead ECGs to compute the QRS duration. This in silico trial showed that, in the presence of proximal LBBB, ventricular synchrony was improved more significantly by HBP compared to BVP, and that LBBP alone without AV delay optimisation leads to sub-optimal response due to prolonged RV activation. In patients with right bundle branch intrinsic activation, AV delay optimisation allows the stimulus to travel along the conducting right bundle branch leading to fast RV activation, while the LV remains synchronised by the LBBP stimulus. Although baseline LBBB activation metrics and ECGs morphology were validated using values and characteristic available in the literature reported for LBBB patients, response to pacing predicted by the model was not validated. The authors also did not consider conduction disturbances other than proximal LBBB, making the study conclusions valid only for a subset of patients with an indication for cardiac physiologic pacing. Furthermore, a reaction-Eikonal model, while it is computationally cheaper, is a simplification of the propagation of the electrical stimulus in cardiac tissue compared to more complex and accurate monodomain or bidomain formulations. Zhu et al. [40] also compared different CSP delivery modes using a bidomain model of a rabbit heart to represent cardiac tissue activation. Using this model, the authors simulated baseline LBBB, HBP and LBBP with and without AV delay. Their results agreed with the study by Strocchi et al. [41], additionally providing information about how the 12-lead ECG changes in response to different forms of CSP. In this study, the ECG morphology simulated during LBBB on the rabbit model was compared with an ECG from a LBBB patient during baseline and HBP, showing that the model predicts QRS duration reduction similarly to that recorded in the LBBB patient. However, the activation times were not validated, and the authors compared the results from an animal model with human data. Moreover, as opposed to Strocchi et al. where the effect of heart shape was accounted for by using twenty-four patient-specific heart anatomies, Zhu et al. used only one rabbit geometry. This modelling study, consistently with others, assumed that LBBB correction led to a healthy ECG, therefore not considering a scenario where LBBB is not the only conduction disturbance. Although the studies presented by Strocchi et al. and by Zhu et al. provided insight into response to different types of physiologic pacing, it is important to point out that these findings apply only in the presence of LBBB. Indeed, response to pacing depends on several often patient-specific features, including LV geometry, type of ventricular conduction delay and the presence of scar, that were not accounted for in these in silico studies.

The conclusions of the in silico studies by Strocchi et al. and Zhu et al. were subsequently confirmed by clinical trials comparing BVP and CSP [44–51]. In 2023, Ali et al. [44] used QRS duration, electrocardiographic imaging (ECGi) and acute haemodynamic response to compare BVP, HBP and LBBAP. HBP and LBBAP achieved better ventricular electrical synchrony compared to BVP, and LV activation times were comparable between HBP and LBBAP. However, as reported by the authors, HBP led to shorter biventricular activation times than LBBAP, due to prolonged RV activation times during LBBAP. In a third of the patients analysed in this study, successful AV delay optimisation during LBBAP was possible thanks to the presence of favourable RV intrinsic activation through the right bundle, which led to fusion with the paced activation from the left bundle and therefore improved RV activation. In the remaining two thirds, AV delay optimisation was not successful due to heart block or atrial fibrillation, or the patients already presented multiple RV activation wavefronts during LBBAP without optimised AV delay. These results constitute a validation of the in silico studies presented above. Several small non-randomised studies also consistently report better synchrony with HBP and LBBAP compared with BVP [44–48, 50]. Published results from randomised clinical trials looking at mid- to long-term responses however concluded that CSP is either better or comparable to BVP at 6 months to 1 year follow-up [48, 52–56] indicating the need to further investigate the factors affecting response to CSP. Nevertheless, the in silico studies by Strocchi et al. and Zhu et al. constitute an example of how computational models can be used to investigate mechanisms underlying response to CSP, and therefore to guide clinical trials towards response optimisation.

All studies described so far only accounted for electrical synchrony, discarding how this translates into improved mechanical synchrony and cardiac function. To account for mechanical synchrony in their model, Strocchi et al. extended their electrophysiology model [41] to a three-dimensional mechanics model to investigate mechanical response to CSP [57]. In particular, septal motion was quantified to determine if HBP and LBBP correct for septal flash, a right to left dyssynchronous septal motion observed in patients with LV dyssynchrony during early ventricular systole. The study showed that, while HBP completely eliminates septal flash arising from LBBB, LBBP without optimised AV delay leads to abnormal left to right septal motion due to RV late activation. Although this study was able to correlate electrical with mechanical synchrony resulting from CSP, proximal LBBB was the only pathology considered in the study, therefore limiting its conclusions to patients where LBBB leads to septal flash. Furthermore, due to the computational cost of cardiac mechanics, the simulations accounted for only four geometries, likely not capturing the anatomical variability within the heart failure population. A more computationally efficient modelling approach to simulate local mechanics and haemodynamics is to employ lumped parameter models. Meiburg et al. [58] used CircAdapt, a lumped parameter model for the whole circulatory system, to simulate local strains and myocardial work distribution within the ventricles in response to RV pacing, BVP and HBP in patients with normal cardiac function. The model predicted that HBP leads to the most homogeneous strains, while selective LBBP significantly increased RV load. Non-selective LBBP mitigates this phenomenon but increases LV strain heterogeneity. This study assumed healthy cardiac tissue and function at baseline and did not consider the presence of scar or abnormal baseline activation such as LBBB. Therefore, these findings cannot be applied to patients with failing hearts due to the baseline impaired cardiac function and strain distribution, which are commonly found in patients with an indication for pacing. Although neither of these mechanical models provided validation against experimental or clinical data nor were able to provide insights into long-term response to CSP, they constitute promising examples of how in silico electromechanics models can enrich the spectrum of information provided by computational models, making them even appealing to the clinical community for a wider range of clinical applications.

The computational studies described above accounted for a limited range of pathologies, mostly proximal LBBB and/or AV block, leading to favourable outcomes for CSP. However, clinical trials investigating response to CSP report success rates as low as 54% [59] and 78% [60] for HBP and LBBP, respectively, depending on the experience of the implanter and on the patient’s underlying pathologies. This indicates the need to better differentiate patients who would or would not benefit from CSP to avoid unnecessarily difficult implantations and to reduce the costs associated with heart failure patients’ management. In this scenario, in silico trials can be used to consistently investigate how response to CSP is affected by different pathologies. Strocchi et al. [61] used a cohort of twenty-four heart anatomies and an Eikonal model to simulate ventricular activation during proximal LBBB, BVP, HBP and LBBP. Then, different conduction disturbances were introduced (for example diffuse LV conduction disease or the presence of septal scar) and changes in response to pacing were quantified. Baseline LBBB activation as well as electrical response to BVP and HBP were validated against in-house and literature ECGi data collected from LBBB patients, showing that the model was able to replicate features of LBBB activation and observed acute electrical clinical response to pacing. However, changes in ECG due to different conduction diseases or pacing delivery methods were not included in the study. The authors concluded that patients with LV diffuse conduction disease are less likely to respond to CSP alone, and that an additional LV epicardial lead to deliver HOT-CRT and LOT-CRT achieves better overall synchrony. On the other hand, in the presence of septal scar, CSP is completely ineffective, unless the His-Purkinje system remains viable in the sub-endocardial layer overlapping with the septal scar. These conclusions agree with the results reported by Upadhyay et al. [62], where the authors showed that LBBB correction through HBP could be achieved in patients with proximal LBBB but not with diffuse conduction disease. In a similar in silico study using the same model, response to BVP and CSP was investigated in RBBB patients [63]. The simulated activation during RBBB alone and with anterior or posterior LV fascicular block was validated against literature activation maps recorded in RBBB patients. Quantitative validation was provided by comparing activation metrics simulated during RBB against literature values measured in RBBB patients. In this case, modelling was used to test whether septal capture through non-selective LBBP or anodal capture on the RV side of the septum led to RBBB correction during LBBP, as hypothesised by Vijayaraman et al. [64]. The results of this in silico study showed that non-selective capture was unable to explain RBBB correction and that anodal capture of the RV myocardium improved response but still could not correct the RBBB. Complete correction could be achieved in the model only when the anodal stimulus captured the right bundle, leading to synchronous activation of the ventricles. The in silico model by Strocchi et al. was finally used to test the efficacy of the EBR leadless pacing system in response to different pacing settings in the presence of proximal LBBB [65]: location of the LV lead (left bundle vs lateral wall), location of the RV lead (RV apex vs septum), RV-LV delay and type of left bundle capture. The baseline LBBB model was validated against in-house ECGi data collected from LBBB patients, showing that the model was able to replicate clinically measured activation pattern and activation metrics. The results showed that leadless LV lateral wall pacing is less sensitive than leadless LBBP to prolonged RV-LV delays. Response to leadless LBBP was worsened by RV septal vs apical pacing and by myocardial vs selective or non-selective left bundle capture. This in silico trial shows how computational models can be used to noninvasively test new pacing technologies and to explore which pacing settings are more likely to improve and optimise response. Ultimately, these results can help guiding clinical trials in order to reduce required procedure times to optimise response to pacing.

## Future Directions

The in silico studies described above show that computational models of the heart have a role to play in improving response to CSP. However, due to the timescales currently required to generate patient-specific anatomical models and/or to run computationally expensive simulations, insilico models are still not incorporated in a clinical setting. The development of more efficient numerical methods can reduce the time required to perform heart simulations. Whole-heart electrophysiology models, such as the reaction-Eikonal equations, can be solved in a few seconds on a desktop, making them potentially compatible with clinical time scales. These simulations can provide activation times and transmembrane voltage propagation and may be suitable for exploring the effect of pacing on heart electrical excitation, but some applications might still require more computationally demanding monodomain or bidomain solves. Cardiac computational mechanics is less well developed than electrophysiology, it is more computationally expensive, and the simulations are less stable. For this reason, more advanced modelling approaches, such as Gaussian processes emulators [66] or neural networks [67], are currently required to achieve the necessary speed up to incorporate mechanics within clinical workflows. Once computational modelling timescales have matched clinical timescales, heart models of varying complexity could be used to investigate different aspects of response to CSP. For instance, computational electrophysiology models could be used to optimise HBP lead position before or during the implantation procedure, potentially reducing fluoroscopy times, patient risk and unsuccessful implantations. Similarly, as discussed above, the depth of the LBBAP lead determines the type of capture, but it is unclear when selective pacing might be required or LV septal pacing might suffice in order to achieve patient response. Different types of capture could therefore be tested in silico prior to the implantation procedure to prevent the need to test multiple pacing thresholds and locations in vivo.

Patient stratification and selection for CSP delivery is challenging, due to the highly heterogeneity of the heart failure patient population, where dyssynchrony could be caused by a plethora of conduction disturbances. In some of the studies presented above, in silico trials were used to investigate which conduction disturbances are likely to be corrected with CSP rather than BVP, indicating that a patient-specific approach might be required. In this context, patient-specific models, where model parameters are tailored to a single patient, might provide a noninvasive framework to detect which conduction disturbances are causing the dyssynchrony in the patient’s heart and to determine whether that specific patient might respond to CSP. However, current calibration techniques are still time-consuming and require a wide range of clinical data, making personalised in silico models currently out of reach for clinical practice.

All in silico models presented in this review account for acute electrical or mechanical response and were unable to provide information about long-term effects of CSP. Mechanical models can however be used to predict remodelling after pacing. Oomen et al. [68] used a lumped parameter framework coupled with a growth model to predict pacing-induced reverse remodelling by simulating myocyte adaptation to changes in local fibre strains in an ischemic canine model. The model was able to predict short and long-term changes in response to CRT and showed that reverse remodelling following CRT is dependent on pacing location and does not always correlate with QRS duration. In the future, growth models could be used to compare long-term remodelling in heart failure patients following BVP and CSP and to investigate whether the acute improvements induced by CSP also lead to long-term benefits for the patients.

The in silico studies presented in this review demonstrate the maturity of cardiac modelling and simulation, and how in silico trials can complement and inform the development and evaluation of cardiac device therapies. The cohorts of virtual models were able to prospectively predict the outcomes of clinical studies and provide a case study on the growing role of in silico trials in designing and interpreting clinical studies of novel device therapies. These studies support the earlier and wider use of in silico studies in the development of novel heart failure device therapies, in line with changes in regulatory standards [69].

## Conclusion

CSP is an emerging delivery method for pacing. However, before CSP is adopted in standard clinical practice, large, randomised, multi-centre clinical trials are needed to investigate its safety and long-term outcomes compared to BVP. In silico studies and trials can accelerate this process by noninvasively testing new technologies, identifying patients that are likely to respond to CSP, and helping understand the mechanisms behind optimal CSP delivery while avoiding device battery draining. However, clear and in-depth model validation procedures are needed to increase confidence and reliability of in-silico studies results.

### Supplementary Information

ESM 1

## Abbreviations

CRT: Cardiac resynchronisation therapy
BVP: Biventricular pacing
RV: Right ventricle
LV: Left ventricle
LBBB: Left bundle branch block
QRSd: QRS duration
LVEF: Left ventricular ejection fraction
CSP: Conduction system pacing
HBP: His bundle pacing
LBBP: Left bundle branch pacing
LBBAP: Left bundle branch area pacing
HOT-CRT: His optimised cardiac resynchronisation therapy
LOT-CRT: LBBP optimised cardiac resynchronisation therapy
ECGi: Electro-cardiographic imaging
