# Supplementary material for: Computational Modelling Enabling In Silico Trials for Cardiac Physiologic Pacing
Source: J Cardiovasc Transl Res. 2023 Oct 23;17(3):685–94. doi: 10.1007/s12265-023-10453-y (PMC11219462; doi:10.1007/s12265-023-10453-y)
Supplement: Supplementary file 1 — ESM 1 [file 12265_2023_10453_MOESM1_ESM.docx]

| **Reference** | **Modelling Approach** | **Type of Geometry** | **Type of Pacing** | **Validation** | **Conduction Abnormality** | **Baseline LV function** | **Scar** | **Study Conclusion** |
| --- | --- | --- | --- | --- | --- | --- | --- | --- |
| **Lead positioning for CSP delivery** | | | | | | | | |
| *Vigmond et al*^1^ | Electrophysiology, bidomain | Simplified | HBP | - | Not applicable | Not applicable | No | Increasing distance of the lead from the His increases pacing threshold |
| *Barone et a*l^2^ | Electrophysiology, monodomain | Simplified and human biventricular + torso | HBP | ECG morphology validated against literature data | Complete AV block or complete and incomplete LBBB | Not applicable | No | HBP lead should be placed perpendicular to the His. Angled positions lead to increased pacing thresholds |
| **Comparison between different pacing modalities** | | | | | | | | |
| *Strocchi et al*^3^ | Electrophysiology, reaction Eikonal | Cohort of 24 human failing hearts | CRT, HBP, LBBP | Baseline activation and ECG morphology validated against literature data | Proximal LBBB | Not applicable | No | CSP achieves better synchrony than CRT; AVD optimisation is needed for LBBP to be comparable to HBP |
| *Zhu et al*^4^ | Electrophysiology, bidomain | Rabbit biventricular | HBP, LBBP | ECG morphology validated against ECG from a LBBB patient | LBBB | Not applicable | No | AVD delay optimisation leads to comparable response between HBP and LBBP |
| *Strocchi et al*^5^ | Three-dimensional electromechanics | Cohort of 4 human failing hearts | HBP, LBBP | - | Proximal LBBB | Abnormal septal motion (septal flash) | No | HBP corrects septal flash, while LBBP without AVD optimisation does not |
| *Meiburg et al*^6^ | Three-dimensional electrophysiology; zero-dimensional circulatory system | Biventricular healthy average model (for electrophysiology) | RVP, HBP, LBBP | - | Not reported | Not reported | No | HBP leads to uniform strains, while LBBP increases RV load |
| *Strocchi et al*^7^ | Electrophysiology, reaction Eikonal | Cohort of 24 human failing hearts | CRT, HBP, LBBP, HOT-CRT, LOT-CRT | Baseline and paced metrics validated against human ECGi data | Proximal LBBB +  LV diffuse conduction disease, septal/lateral scar, slow myocardium | Not applicable | LV Septal or Lateral | Diffuse LV conduction benefits from HOT-CRT or LOT-CRT but not CSP alone; CSP is ineffective with septal scar |
| *Strocchi et al*^8^ | Electrophysiology, reaction Eikonal | Cohort of 24 human failing hearts | CRT, HBP, LBBP, LBBP + anodal RV capture | Baseline activation and metrics validated against human literature data | Proximal RBBB + LAFB, LPFB, LV or RV diffuse conduction disease, slow myocardium | Not applicable | No | LBBP + anodal RV capture leads to improved RV activation only if the right bundle is anodally stimulated |
| *Strocchi et al*^9^ | Electrophysiology, reaction Eikonal | Cohort of 24 human failing hearts | Leadless LBBP or LV lateral wall pacing | Baseline activation pattern and metrics validated against human ECGi data | Proximal LBBB | Not applicable | No | Leadless lateral pacing is less sensitive to prolonged RV-LV delays; RV septal pacing is worse than apical for leadless LBBP |

**Table 1. Summary of computational models for cardiac physiologic pacing.** This table summarises the studies included in the review. The columns describe (from the left): reference to the study, which type of modelling approach the study used, the type of geometry, which pacing modalities the study investigated, if and how the model was validated and which type of human or animal data were used, which conduction abnormalities were considered, what type of baseline LV function, if scar was considered and finally the main study conclusion. Abbreviations: His bundle pacing (HBP), AV (atrioventricular), cardiac resynchronisation therapy (CRT), left bundle branch pacing (LBBP), left bundle branch block (LBBB), atrioventricular delay (AVD), right ventricular pacing (RVP), right ventricle (RV), left ventricle (LV), His-optimised CRT (HOT-CRT), LBBP-optimised CRT (LOT-CRT), conduction system pacing (CSP), electrocardiograhic imaging (ECGi).

# References

1. Vigmond EJ, Neic A, Blauer J, Swenson D, Plank G. How Electrode Position Affects Selective His Bundle Capture: A Modelling Study. *IEEE Trans Biomed Eng*. 2021;68(11). doi:10.1109/TBME.2021.3072334

2. Barone A, Grieco D, Gizzi A, et al. A Simulation Study of the Effects of His Bundle Pacing in Left Bundle Branch Block: Simulation of His Bundle Pacing in Left Bundle Branch Block. *Med Eng Phys*. 2022;107. doi:10.1016/j.medengphy.2022.103847

3. Strocchi M, Lee AWC, Neic A, et al. His-bundle and left bundle pacing with optimized atrioventricular delay achieve superior electrical synchrony over endocardial and epicardial pacing in left bundle branch block patients. *Heart Rhythm*. 2020;17(11). doi:10.1016/j.hrthm.2020.06.028

4. Zhu H, Jin L, Huang Y, Wu X. A computer simulation research of two types of cardiac physiological pacing. *Applied Sciences (Switzerland)*. 2021;11(1). doi:10.3390/app11010449

5. Strocchi M, Neic A, Gsell MAF, et al. His Bundle Pacing but not Left Bundle Pacing Corrects Septal Flash in Left Bundle Branch Block Patients. In: *Computing in Cardiology*. Vol 2020-September. ; 2020. doi:10.22489/CinC.2020.030

6. Meiburg R, Rijks JHJ, Beela AS, et al. Comparison of novel ventricular pacing strategies using an electro-mechanical simulation platform. *Europace*. 2023;25(6). doi:10.1093/europace/euad144

7. Strocchi M, Gillette KK, Neic A, et al. The effect of Scar and His-Purkinje and Myocardium Conduction on Response to Conduction System Pacing. *Journal of the American College of Cardiology: Clinical Electrophysiology (Under review)*. Published online 2022.

8. Strocchi M, Gillette K, Neic A, et al. Comparison between conduction system pacing and cardiac resynchronization therapy in right bundle branch block patients. *Front Physiol*. 2022;13. doi:10.3389/fphys.2022.1011566

9. Strocchi M, Wijesuriya N, Elliott MK, et al. Leadless biventricular left bundle and endocardial lateral wall pacing versus left bundle only pacing in left bundle branch block patients. *Front Physiol*. 2022;13. doi:10.3389/fphys.2022.1049214
